# Rapid spread of OXA-244-producing *Escherichia coli* ST38 in Germany: insights from an integrated molecular surveillance approach; 2017 to January 2020

**DOI:** 10.2807/1560-7917.ES.2020.25.25.2000923

**Published:** 2020-06-25

**Authors:** Katrin Kremer, Rolf Kramer, Bernd Neumann, Sebastian Haller, Niels Pfennigwerth, Guido Werner, Sören Gatermann, Horst Schroten, Tim Eckmanns, Jörg B Hans

**Affiliations:** 1 Robert Koch-Institute, Department for Infectious Disease Epidemiology, Berlin, Germany; 2 Postgraduate Training for Applied Epidemiology (PAE), Robert Koch-Institute, Berlin, Germany; 3 These authors contributed equally to this manuscript; 4 Robert Koch Institute, Division of Nosocomial Pathogens and Antibiotic Resistance, Wernigerode, Germany; 5 National Reference Centre for multidrug-resistant Gram-negative bacteria, Ruhr University Bochum, Bochum, Germany; 6 Department of Pediatrics, Pediatric Infectious Diseases, Medical Faculty Mannheim, Heidelberg University, Mannheim, Germany

**Keywords:** Carbapenemase, Enterobacterales, whole genome sequencing, WGS

## Abstract

Annually, increasing numbers of OXA-244-producing *Escherichia coli* in 13 German federal states prompted us to initiate an outbreak investigation. Whole genome sequencing revealed that among 148 isolates analysed, most belonged to sequence type 38 with 62 isolates forming a genetically distinct cluster. Although no epidemiological link could be identified between cases, ongoing investigations suggest non-healthcare associated transmission. A screening-PCR was developed facilitating early detection of ST38 cluster isolates to identify the source and transmission route.

A rise in detection of community-acquired infections with OXA-244-carbapenemase-producing *Escherichia coli* in at least nine European countries prompted a rapid risk assessment (RRA) by the European Centre for Disease Prevention and Control (ECDC) in the beginning of 2020 [1]. The rise in detection was deemed worrisome as despite of challenging detection of the chromosomally-encoded carbapenemase, a rapid and simultaneous increase was found. Genetically distinct clusters, predominantly of sequence type (ST) 38 were identified, thus a common source and a transmission via contaminated food products could not be ruled out. Germany reported the highest numbers of detected isolates among the affected countries.

Here we provide details on our integrated molecular surveillance (IMS) results and consecutive outbreak investigations in Germany that led to the RRA. Our IMS involved molecular investigation via whole genome sequencing (WGS) to identify relatedness of isolates, followed by epidemiological investigations of linked notified carbapenem-resistant *E. coli* cases to understand modes of transmission and to identify possible sources. A newly developed screening-PCR will facilitate rapid detection of the ST38 cluster isolates.

## Molecular investigation of OXA-244-producing *Escherichia coli*

**Figure 1:**
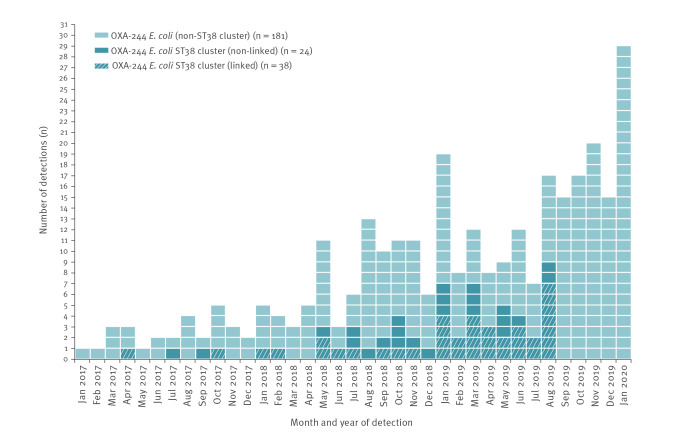
Number of detected OXA-244-producing *Escherichia coli* isolates by month, Germany, January 2017–January 2020 (n = 243)

Between January 2017 and the end of February 2019, a total of 144 OXA-244-producing *E. coli* isolates were identified at the German National Reference Centre for multidrug-resistant Gram-negative bacteria (NRC) in Bochum (Figure 1), of which 86 isolates with information on hospital origin or diagnostic laboratory were subjected to Illumina (Illumina, San Diego, United States) WGS. As continued increase was noted and preliminary results pointed towards an ongoing nationwide spread [2], our analysis focused on most recent OXA-244-producing *E. coli* isolates from March 2019 onwards including those with only partially complete metadata.

ST: sequence type.

Respective monthly numbers of ST38 cluster isolates as well as linked cases are shown. Sequencing data and information on STs was not available for isolates from September 2019 onwards.

**Figure 2:**
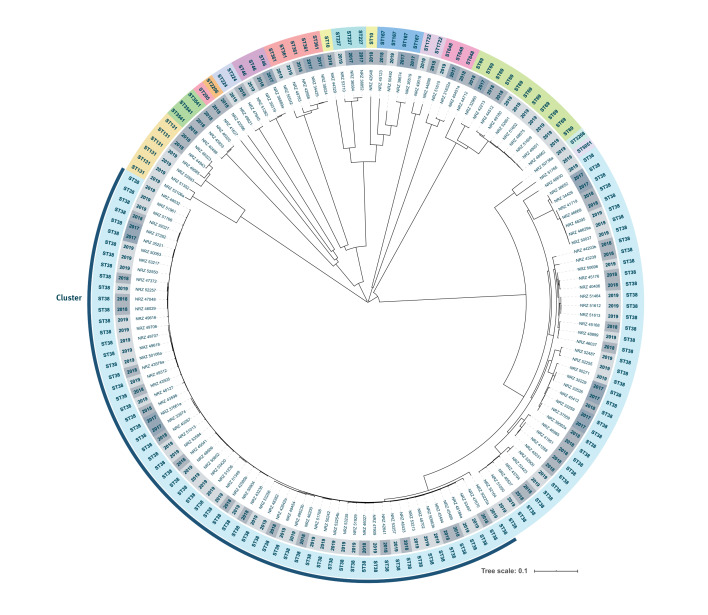
Phylogenetic tree of core genome multilocus sequence typed OXA-244-producing *Escherichia coli* isolates, Germany, January 2017­­–August 2019 (n = 148)

Until the end of August 2019, 152 isolates were subjected to WGS with 148 isolates having sufficient sequence data for further analyses which included in silico multilocus sequence typing (MLST) and core genome (cg)MLST using the *E. coli* scheme (2,513 loci) as implemented in the SeqSphere+  software version 6.0.0 (Ridom, Muenster, Germany). Among the 148 isolates analysed, we identified 16 different sequence types (ST), with a dominance of ST38 (n = 100). Results of cgMLST revealed close genetic relatedness of ST38 isolates, particularly among 62 isolates forming a distinct cluster with a maximum of 27 allelic differences among core genes, and a distance of > 118 alleles to other ST38 isolates (Figure 2). By the end of January 2020, the NRC had identified a total of 243 OXA-244-producing *E. coli* isolates indicating a continuous spread in Germany (Figure 1).

ST: sequence type.

SeqSphere+ software version 6.0.0 (Ridom, Muenster, Germany)  was used to determine an allelic profile and used to calculate a neighbour-joining phylogenetic tree. Metadata were annotated using Interactive Tree Of Life [18]. Year of isolation is shown as inner greyish circle whereas the 16 different ST are colour-coded in the outer circle. Highlighted in blue is the cluster of ST38 isolates (n = 62).

## Epidemiological investigation

**Figure 3:**
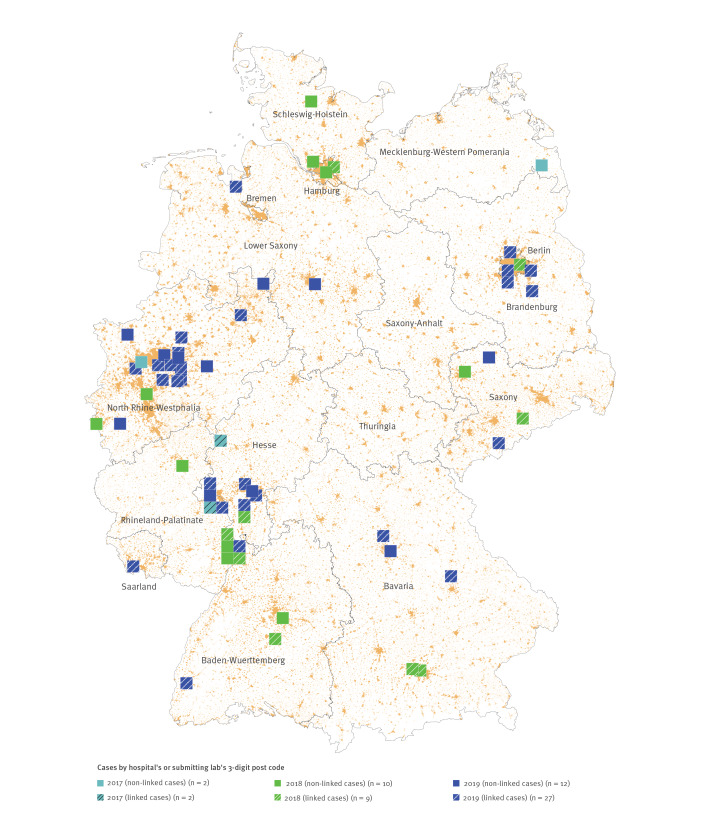
Distribution of cases with OXA-244-producing *Escherichia coli* ST38 isolates by year and notification status, Germany, January 2017–January 2020 (n = 62)

To infer public health implications of closely related isolates by WGS, additional investigations for epidemiological association are required [3]. Our epidemiological investigation focused on the predominant cluster of 62 OXA-244-producing *E. coli* ST38 isolates. Isolates from cases were mapped based on the postal code of hospital of origin (where available) or diagnostic laboratory, revealing a supra-regional dissemination over 13 of the 16 German federal states (Figure 3) without a discernible pattern. Available information (specimen type, date of detection and patient age) for each isolate was extracted from the anonymized NRC database and used for linking with a second data set. In 2016 Germany has introduced a mandatory surveillance for colonisation or infection with carbapenem-resistant Enterobacterales and *Acinetobacter* spp.. We linked the available laboratory data with information from the national electronic reporting system for notifiable diseases. This enabled us to obtain additional case information and to identify the responsible health authority. Overall, 38 of 62 isolates could be linked (Figure 3). For these linked cases, additional personal data were available: sex, infection/colonisation ratio and age (age median: 56 years; age range: 0–82 years) were equally distributed (Table 1). All responsible public health authorities of affected German federal states were individually notified in August and October 2019. Together with the local public health authorities, we collected further case information via an exploratory questionnaire (Supplement 1) (response rate: 24/38, 63%), as summarised in Table 1. In the following all available information is summarised, the denominator varies as missing information was not counted. Of cases with available information, briefly, 27 of 36 cases were hospitalised at the time of detection of OXA-244-producing *E. coli*; 14 of 21 cases had a history of hospitalisation before their current hospital stay; eight of 18 cases had an underlying disease. Although nine of 14 cases had a stay abroad within 12 months before detection, no geographical areas with predominance were identified. Interestingly, half of the cases were detected via hospital admission screening or at outpatient treatment (12/24). These findings, and the absence of any common healthcare-related exposure or person-to-person transmission for closely related isolates, pointed towards transmissions outside healthcare settings.

NRC: German National Reference Centre for multidrug-resistant Gram-negative bacteria.

Year of isolate receipt at NRC are colour-coded in blue. Notified cases are labelled with diagonal stripes. Cases are mapped by 3-digit postcode of the sample collection locality (hospital) or alternatively of reporting laboratory. Background colour (orange) indicates population density.

**Table 1:** Case characteristics from notification and National Reference Centre for multidrug-resistant Gram-negative bacteria data as well as results of exploratory questionnaires and interviews, Germany, January 2017–January 2020 (n = 62)

Characteristics	Total cases (n = 62)	Non-linked cases (n = 24)	Linked cases (n = 38)^a^	Interviewed linked cases (n = 6)
**Sex**				
Male	17	NA	17	3
Female	21	NA	21	3
Unknown	24	24	0	0
**Median age (years) (range)**	55 (0–86)	49.5 (0–86)	56 (0–82)	24 (0–62)
**Age groups (years)**				
< 3	9	3	6	1
3–17	6	1	5	1
18–39	10	6	4	2
40–64	16	5	11	2
≥ 65	19	7	12	0
Unknown	2	2	0	0
**Material of isolate**				
Rectal	27	10	17	2
Urine	20	7	13	3
Blood	8	4	4	0
Intraabdominal	1	0	1	1
Respiratory	1	0	1	0
Wound	1	0	1	0
Other	4	3	1	0
**Status of infection**				
Colonised	Unknown^b^	NA	12	4
Infected	Unknown^b^	NA	11	2
Unknown	Unknown^b^	NA	15	0
**Hospitalisation at detection,** Yes^c^	Unknown^b^	NA	27/36	4
**Previous Hospitalisation (< 12 months),** Yes^c^	Unknown^b^	NA	14/21	4
**Detection via admission screening or outpatient treatment,** Yes^c^	Unknown^b^	NA	12/24	3
**Underlying disease,** Yes^c^	Unknown^b^	NA	8/18	1
**Travel history (< 12 months),** Yes^c^	Unknown^b^	NA	9/14	4
**Contacts to farm animals (< 12 months),** Yes^c^	Unknown^b^	NA	1/13	1
**Vegetarian or vegan diet,** Yes^c^	Unknown^b^	NA	Unknown^d^	1

NA: not available.

^a^ Some of the information for linked cases is from the notification data (n = 38) with additional information coming from the exploratory questionnaire (n = 24).

^b^ Information was only available from linked cases such that providing a total count for all cases was not possible.

^c^ Denominator represents all cases with available information.

^d ^Information on vegetarian or vegan diet was only obtained through trawling questionnaire and thus only available from interviewed linked cases.

Therefore, we further investigated community-related modes of transmission through a trawling questionnaire, interviewing the most recent notified cases to limit recall error by delay of interview [4] (Supplement 2). In six interviews, no common diet or common exposures could be identified among the cases, but in all households meat was processed and consumed. (Table 1).

## International alert

Since the spread still continued, on 20 December 2019, our major findings were internationally shared via the European Early Warning and Response System (EWRS) and Epidemic Intelligence Information System (EPIS) of the ECDC. This led to the aforementioned ECDC RRA and identification of spread in further European countries [1].

## Screening PCR for the identification of OXA-244 *Escherichia coli* ST38 cluster isolates

**Figure 4:**
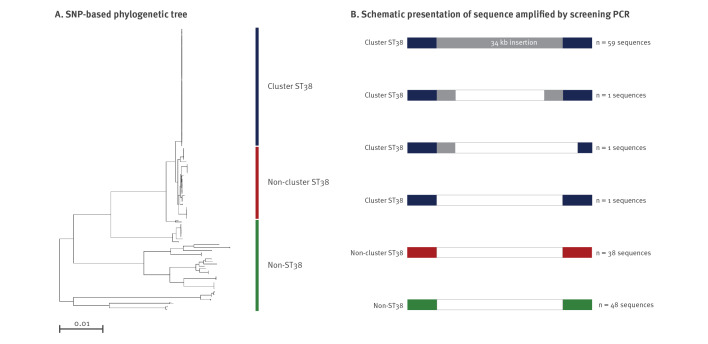
SNP analyses based on core genome multilocus sequence typing results OXA-244-producing *Escherichia coli* ST38 isolates (n = 148)

To accomplish early collection of patients’ epidemiological information for the identification of potential sources and routes of transmission, we developed a PCR assay specific for OXA-244-producing *E. coli* ST38 cluster isolates enabling nearly real-time screening. For this, we performed SNP analyses as implemented in Enterobase [5] to identify genetic differences of isolates. Using the assembly with the highest N50 value of a ST38 cluster isolate as reference genome, SNP analyses based on a total of 227,099 variable sites confirmed cgMLST results (Figure 4). Comparison of alignments revealed a ca 34 kb insertion that was carried only by ST38 cluster isolates (Figure 4) corresponding to an intact Mu-like bacteriophage, as annotated by rapid annotations using subsystems technology (RAST) and PHAge Search Tool Enhanced Release (PHASTER) analyses [6,7]. However, this segment was completely lacking in one isolate, whereas in two other isolates the prophage was not fully integrated but sequences upstream of the transposase gene were present (Figure 4). Based on the phage sequences found in 61 of the 62 ST38 cluster isolates, a specific PCR assay was designed to amplify a 514-bp-fragment partially encompassing the gene that encodes the repressor protein CI (Table 2). WGS results for 144 of the 148 isolates analysed indicated a high concordance rate (97.3%). However, two false positives (specificity 98.6%) as well as two false negatives (sensitivity 98.6%) were observed in our sample collection including the ST38 cluster isolate for which absence of the prophage was known. It has to be noted that the screening approach described here might only be valid for *E. coli* isolates known to carry the *bla*_OXA-244_, as ongoing analyses suggest that the prophage is also integrated in other ST38 contexts.

Neighbour-joining tree based on the SNP analysis of 148 OXA-244-producing E. coli isolates and corresponding schematic sequence alignments. SNP analysis was conducted in Enterobase [5].

Panel A: Cluster ST38, non-cluster ST38 and non-ST38 isolates are represented by the blue, red and green bars, respectively.

Panel B: Schematic representation of sequences with the insertion highlighted in grey identified in cluster ST38 isolates shown in blue. Absence of the insertion was found in non-cluster ST38 and non-ST38 isolates shown in red and green, respectively (see text for details). Different colours represent homologous sequences.

**Table 2:** PCR screening assay for the identification of *Escherichia coli* ST38 cluster isolates

Primer sequences 5’ to 3’	PCR reaction condition^a^
Forward TGAAACGCGAAAGATTGTTG	95 °C 5min; 95 °C 30 s, 58 °C 30 s, 72 °C 30 sec; 35 cycles
Reverse TCAATCGCCCATACAGTTGA	

^a^ In 25 µl containing: 1.8 mM MgCl, 200 μM dNTPs, 1 μM primers (each), 1.25 U AmpliTaq 360 Polymerase and 1 x AmpliTaq 360 Buffer (Thermo Fisher Scientific, Waltham, Massachusetts, United States).

## Ethics

A formal ethical review was not required for the outbreak investigation in accordance with Article 25, Section 1 of the German Infection Protection Act of 2001.

## Discussion

Here, we used for the first time a systematic IMS approach for carbapenemase-producing Enterobacterales (CPE) in Germany by combining WGS data and epidemiological analyses on a national scale. Our results provide further evidence for the rapid and ongoing spread of *bla*_OXA-244_ [1,8]. In Germany, the spread was predominantly driven by genetically-clustered ST38 *E. coli*. Although instances of sporadic nosocomial infections cannot be excluded, it is unlikely that overall patterns reflect sustained healthcare-driven transmissions on a supra-regional scale. Instead, available data suggest that infections are due to non-hospital-acquired transmissions as most cases were detected upon admission or during outpatient treatment. Notably in this regard, younger age groups were more affected in comparison to overall notifications of CRE in Germany: nearly half of cases were below 50 years compared with 18% of all CRE of which 91% were hospitalised in 2018 [9].

Although, worldwide, OXA-244-producing *E. coli* were detected in cases in Columbia [10] or cases were reported with epidemiological links to Indonesia [11], our investigations revealed no association with travel. Furthermore, no direct links to animals or certain food items were found. However, specific food products with a broad dissemination might be one possible vehicle to explain global spread. Carbapenemases were previously detected in various Enterobacterales species from food, livestock and companion animals in Germany and other European countries, although *bla*_OXA-244_ was not identified [12,13]. ST38 is known to be abundant in livestock, such as poultry, and has also been identified along the food production chain [14,15]. Although results of the trawling questionnaire remained ambiguous, food might be a possible vehicle. Ongoing molecular surveillance might reveal seasonal patterns in transmission risk thus enabling to narrow down potential sources of infection. Collaborating with food safety authorities, screening for clustered isolates should be considered and further interviews conducted accordingly. Multiple transmission hypotheses can be considered regarding the spread of OXA-244-producing *E. coli* isolates. The wide dissemination of these isolates in Europe [10,16,17] demonstrates the urgent need for additional investigations including additional data sources as well as enhanced screening capabilities [3].

## Limitations

Isolates predominantly derived from hospitals and cases might not be representative for the underlying mode of transmission. Although this may hinder identification of community exposures, it strengthens the argument of a non-healthcare-related source in the absence of common links. Unknown stability and colonisation period of *E. coli* ST38 in the human gut, results in unknown incubation period, which limited the quality of the epidemiological investigations.

Although sensitivity as well as specificity of the PCR screening assay are high, *bla*_OXA-244_ has to be identified separately and is known be easily missed by phenotypic methods [17].

## Conclusions

The ongoing spread of OXA-244-producing *E. coli* in the community is of great concern. Additional studies are needed to understand the driving forces responsible for the dissemination of *bla*_OXA-244_, which is not exclusively, but primarily driven by genetically-clustered *E. coli* ST38. In the absence of real-time WGS, the here described PCR screening assay may facilitate fast identification of ST38 cluster isolates and allow immediate epidemiological investigations of cases. Further cross-border data sharing and collaboration is needed to identify risk factors for OXA-244-producing *E. coli* colonisation, to investigate potential cross-border transmissions, to guide screenings of at-risk-population in healthcare settings and to guide control measures to stop dissemination in the community.

## Supplementary Data

247000 Supplementary Material
