# Supplementary material for: Rapid spread of OXA-244-producing Escherichia coli ST38 in Germany: insights from an integrated molecular surveillance approach; 2017 to January 2020
Source: Euro Surveill. 2020 Jun 25;25(25):2000923. doi: 10.2807/1560-7917.ES.2020.25.25.2000923 (PMC7331143; doi:10.2807/1560-7917.ES.2020.25.25.2000923)
Supplement: Supplementary Material [file 20-00923_HALLER_OXA_Supplementary_Material.pdf]

Supplementary Material

This supplementary material is hosted by Eurosurveillance as supporting information alongside the article [Rapid spread of OXA-244-producing *Escherichia coli* ST38 in Germany: insights from an integrated molecular surveillance approach; 2017 to January 2020] on behalf of the authors who remain responsible for the accuracy and appropriateness of the content. The same standards for ethics, copyright, attributions and permissions as for the article apply. Supplements are not edited by Eurosurveillance and the journal is not responsible for the maintenance of any links or email addresses provided therein.

Supplement 1

Questions for local health authorities and/or clinics to determine possible common exposures of nationwide detected cases.

Investigations by the Robert Koch Institute on a national outbreak with carbapenem-resistant E.coli.
Confirmed case: *patient with OXA-244 producing E. coli sequence type (ST) 38 belonging to Cluster (colonized or infected)*

1. What diagnoses led to the hospital stay during which a carbapenem-resistant *E.coli* was first detected?

Diagnoses: ____

2. Had the case a surgery before detection of carbapenem-resistant *E.coli* (in the last 12 months)?

yes □ / no □

3. What was the reason to implement this microbiological testing / screening in the hospital?

Screening indication: ____

Did the screening take place on hospital admission?

yes □ / no□

4. Has the case had a hospital stay (> 3 days) in the past 12 months?

yes □ / no □

If yes,

In the same hospital as for carbapenem-resistant E.coli detection?

yes □ / no □

In another hospital in Germany? yes □ / no □

In another hospital outside of Germany? yes □ / no □

If so, where? ____

5. Did the case have contact with patients or shared a room with patients with treated multidrug resistant pathogen infection or colonization?

yes □ / no □

6. Known hospital stay of family members of the case in the same clinic in the past 12 months?

yes □ / no □

7. Known carbapenem-resistant E.coli -evidence in the family of the case?

yes □ / no □

8. Has the case been abroad within the past 12 months?

yes □ / no □

If so, were: ____

9. Does the case have direct contact with animals in livestock farming?

yes □ / no □

10. Do family members have direct contact with livestock animals?

yes □ / no □

11. Does the case have a migration background?

yes □ / no □

If so, where are the family roots?_________________________________

12. Did the case get a special diet in the last 12 months (e.g. food probes, special food, probiotic therapies)

yes □ / no □

If so, which? _________________________________

Supplement 2

Questions for telefon interview of confirmed cases to determine possible common exposures of nationwide detected cases.

Investigations by the Robert Koch Institute on a national outbreak with carbapenem-resistant E.coli.
Confirmed case: patient with OXA-244 producing E. coli sequence type (ST) 38 belonging to Cluster (colonized or infected)

[i] **Y N ? INTRODUCTION QUESTIONS**

☐ ☐ ☐ How many people live in your household?________________________________

☐ ☐ ☐ Had you or any other person in your household previously been found to be resistant to antibiotics?
If yes, who and when?____

☐ ☐ ☐ Has any of your family or friends, who are not part of the household been found to be resistant to antibiotics in the past 12 months?
☐ ☐ ☐ If yes, who and when?________________________________

Have you been in outpatient treatment in the past 12 months?
If yes, due to which illness? ____

☐ ☐ ☐ Have you been hospitalized in the past 12 months?
If yes, due to which illness?____

In which hospital was it and when? ____________________________________

☐ ☐ ☐ _ _ _ _ _ _ _ _ _ _ _ _ _ Have you been hospitalized in the previous 12 months prior to this hospital stay?
If yes, due to which illness?____

In what hospital was that?____

☐ ☐ ☐ Have you been abroad for medical treatment in the past 12 months?
If so, because of which illness? ____
Where?____

☐ ☐ ☐ Have you been in contact with healthcare facilities abroad in the past 12 months?
If so, in what context?____

*When and where?*____

☐ ☐ ☐ Has anyone in your household been hospitalized in the past 12 months?
If so, because of which illness?____________________________________

In what hospital was that?____

ANIMAL CONTACTS

☐ ☐ ☐ Do you have pets or farm animals?
If yes, which?____

☐ ☐ ☐ Have you had contact with farm animals in the past 12 months, e.g. Visit to the farm, petting zoo, professional activity?
If yes, which?____

Y	N	?	**EMPLOYEMENT**
☐	☐	☐	Do you work in a community facility (e.g. day care center, school, nursing home)?
☐	☐	☐	Do you work in healthcare?
			If so, in which area?____
☐	☐	☐	------------To your knowledge, have you cared for patients with proven multi-resistant pathogens?
			**CONTACTS ABROAD**
☐	☐	☐	Have you been abroad in the past 12 months?
			If so, when and where?____
☐	☐	☐	Has anyone else in your household been abroad in the past 12 months?
			If so, who and where?____
☐	☐	☐	Has anyone else in your family or circle of friends been abroad in the past 12 months?
			If so, who and where?____
			**NUTRITIONAL HABITS**
			Is your diet predominantly or exclusively based on
☐	☐	☐	Vegetarian or vegan
☐	☐	☐	According to a religious rule (e.g. kosher, halal)
☐	☐	☐	Uncooked meat (e.g. “Mettwurst”) or raw sausage e.g. Tea-sausage, Salami, “Knacker”, etc.?
			____

[ii] Y	N	?	**POINTS OF CONSUMPTION / EXPOSURE OUT OF HOME**
☐	☐	☐	Fast food restaurants (e.g. McDonald’s, North Sea, Pizza Hut, Subway)
☐	☐	☐	Restaurants
☐	☐	☐	“Döner” or falafel snack
☐	☐	☐	Asian snack
☐	☐	☐	Street snack, snack bar (e.g. “Currywurst” snack point)
☐	☐	☐	Delicatessen / specialty shops
☐	☐	☐	Restaurants / snack bars in shopping centers / airports / train stations
☐	☐	☐	Dining place for lunch (e.g. canteen, cafeteria, cafeteria, hospital canteen, nursing home, senior citizens' lunch table)
☐	☐	☐	Childcare facility / after school / school
☐	☐	☐	Birthday or other private celebration (also eating / cooking together with family / friends)
☐	☐	☐	Hotel, train, coach, cruise ship, ferry

[iii] Y	N	?	**ORIGIN OF FOOD FOR THE HOME**
☐	☐	☐	Supermarket / discounter (e.g. Aldi, Kaufland, Lidl etc.)
			What supermarket?________________________________
			What city is this supermarket in?____
☐	☐	☐	Grocery stores for food from other countries (e.g. Asian shops, Indian, Russian)
☐	☐	☐	Health food store, health food store (e.g. Bio Company, Allnatura, which ones?)
☐	☐	☐	Delicatessen / delicatessen / specialty shops
☐	☐	☐	Market stall, street stall, what bought? ____
☐	☐	☐	Farm
☐	☐	☐	Meat / sausage from private production / slaughter (also slaughtered)
☐	☐	☐	Food brought from abroad (e.g. from travel, neighbors, friends, family)

Y	N	?	**RESTAURANT CATEGORIES**
□	□	□	Asian
□	□	□	_ _ _ _ _Thai
□	□	□	_ _ _ _ _ Vietnamese
□	□	□	_ _ _ _ _ Sushi
□	□	□	_ _ _ _ _Japanese (except sushi)
□	□	□	_ _ _ _ _other
□	□	□	Indian / Pakistani
□	□	□	Arabic / Lebanese
□	□	□	Turkish
□	□	□	Italian
□	□	□	Greek
□	□	□	Balkans (Bulgarian, Croatian etc)
□	□	□	Persian (Iranian, Afghan, etc.)
□	□	□	Spanish
□	□	□	French
□	□	□	German
□	□	□	Mexican / Tex-Mex
□	□	□	Other “international”
□	□	□	Vegetarian or vegan
□	□	□	Steakhouse
□	□	□	Fish restaurant
□	□	□	cafeteria
			Other,
			*If so, which?*________________

[iv]	Y	N	?	**FOREIGN OR OTHER FOOD SPECIALTIES (PASTE, OLIVES, etc.)**
A	□	□	□	polenta
B	□	□	□	Tabbouleh (on request: a Lebanese salad)
C	□	□	□	Chickpea paste (hummus)
D	□	□	□	Sesame paste (Tahina)
E	□	□	□	Eggplant paste (Baba ghanuj)
F	□	□	□	Tapenade (olive, tomato, etc.)
G	□	□	□	Aioli (explain on request: A cold white cream consisting mainly of garlic, olive oil and salt is often served with bread in restaurants)
H	□	□	□	Other pastes / spreads from the market stall / health food store / health food store (e.g. bear's garlic, various pastes)
I	□	□	□	Pickled or dried vegetables (peppers, olives, tomatoes)
J	□	□	□	Bamboo shoots
K	□	□	□	

For parents of a case younger than 2 years old only:
If children under the age of 2 live in the household

[v] Y	N	?	**BABY FOOD**
□	□	□	Has your child received baby food this year (e.g. porridge, milk powder)<br>If so, what?____
